# Alternative stable states in inherently unstable systems

**DOI:** 10.1002/ece3.5944

**Published:** 2019-12-20

**Authors:** David M. Mushet, Owen P. McKenna, Kyle I. McLean

**Affiliations:** ^1^ Northern Prairie Wildlife Research Center U.S. Geological Survey Jamestown ND USA

**Keywords:** alternate stable states, community change, dynamic systems, ecological theory, prairie‐pothole wetlands, state shifts

## Abstract

Alternative stable states are nontransitory states within which communities can exist. However, even highly dynamic communities can be viewed within the framework of stable‐state theory if an appropriate “ecologically relevant” time scale is identified. The ecologically relevant time scale for dynamic systems needs to conform to the amount of time needed for a system's community to complete an entire cycle through its normal range of variation. For some systems, the ecologically relevant period can be relatively short (eg, tidal systems), for others it can be decadal (eg, prairie wetlands). We explore the concept of alternative stable states in unstable systems using the highly dynamic wetland ecosystems of North America's Prairie Pothole Region. The communities in these wetland ecosystems transition through multiple states in response to decadal‐long climate oscillations that cyclically influence ponded‐water depth, permanence, and chemistry. The perspective gained by considering dynamic systems in the context of stable‐state theory allows for an increased understanding of how these systems respond to changing drivers that can push them past tipping points into alternative states. Incorporation of concepts inherent to stable‐state theory has been suggested as a key scientific element upon which to base sustainable environmental management.

## Graphical abstract

Alternative stable states are a set of nontransitory states within which a community can exist. Ideas presented within stable‐state theory can be applied to dynamic systems if the normal range of community variation in response to environmental cycles is considered to be the “stable state.” Prairie‐pothole wetland ecosystems provide an example of alternative stable states within a highly dynamic system.

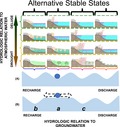

## 1 ALTERNATIVE STABLE STATES

It has long been known by ecologists (eg, Holling, 1973; Lewontin, 1969; Sutherland, 1974; Winberg, 1928) that biological communities can exist in one of several alternative stable states. A “stable state” is a community structure that is nontransitory over an ecologically relevant time scale (Fukami & Nakajima, 2011). Therefore, “alternative stable states” are a set of nontransitory states within which a community can potentially exist. While the stable states themselves are considered to be nontransitory, transitions among alternative stable states can occur and are driven either through changes that occur to state variables internal to a community (eg, shifts in species composition) that move the community about on a fixed environmental landscape, or through changes in the underlying parameters defining the environmental landscape in which the community exists (Beisner, Haydon, & Cuddington, 2003). However, whether through changing community variables or through changing environmental parameters, for a shift to an alternative stable state to occur the driving force of the change must be of sufficient strength to push the system past a tipping point. Once a community is pushed to an alternative stable state, it remains in that new state until another force, either internal or external to the community, acts upon it with sufficient strength to push the community back to its original stable state or to another alternative stable state.

**Figure 1:**
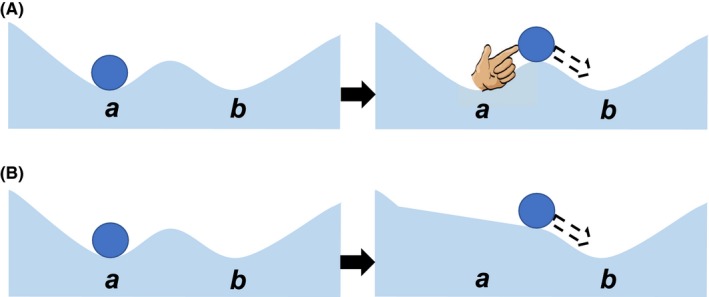
Ball‐and‐cup diagrams depicting how a biotic community (the ball) can respond to changes on internal community variables (A) or external environmental parameters (B). In all situations, the ball will roll “downhill” on the environmental landscape toward stable state at the low points in the environmental landscape (*a* and *b*). Internal drivers are forces within the community itself that cause the community to shift (A). External parameter changes alter the shape of the underlying environmental landscape (B). If of sufficient strength, internal and external drivers can push a community from one stable state (*a*) over a threshold to an alternative stable state (*b*)

The concept of how the two mechanisms of state changes, that is, internal community‐state drivers and external environmental‐parameter drivers, lead to shifts among alternative stable states (Beisner et al., 2003) has often been illustrated heuristically using ball‐in‐cup models (Holling, Schindler, Walker, & Roughgarden, 1995) (Figure 1). In these models, the position of the “ball” represents the current state of the community, while the underlying “cup” represents the environmental landscape within which the community exists. The low areas in the landscape, that is, the bottoms of the cups, are where the stable state exists. The cups themselves are referred to as “domains of attraction.” The depth of the cup is determined by the power of feedback mechanisms that work to keep a community within a cup (ie, a particular state). Without any forces being placed on it, the ball (ie, the community) will naturally move downhill within a domain of attraction toward its stable state at the low point in the cup.

Changes in state variables internal to a community (eg, births and deaths, growth, trophic dynamics) can push the community uphill. If the pressure exerted by a change is great enough to overcome the strength of the feedback mechanisms within the domain of attraction, the community may reach a crest and enter the domain of attraction of an alternative stable state (Figure 1A). Within this new domain of attraction, a new set of forces will then draw the community downhill toward the alternative stable state. Thus, the crest between domains of attraction serves as a tipping point between one stable state and an alternative state. Because history of a system has an influence on ecosystem dynamics, hysteresis, that is, the dependence of system output on its history, can influence when a tipping point is reached and the direction of the resultant shift (Scheffer, Carpenter, Foley, Folke, & Walker, 2001). An example of an internally driven change was demonstrated by Chase (2003) who showed that the community structure and food‐web dynamics of pond snails and snail predators can be different in identical environments and that these alternative states were driven by initial conditions, for example, the order in which species entered the community.

A second mechanism by which the ball could move from one domain of attraction into another is through changes to the underlying environmental landscape, that is, the exogenous drivers (eg, sea‐level rise, storms, and sediment inflows) that act upon a community (Figure 1B). In this case, it is the environmental parameters that are changed. If the change is sufficient such that the ridge separating domains of attraction disappears and the new downward slope is now toward an alternative stable state, the community, responds to the new environment in which it exists and is drawn within the new domain of attraction toward the new low point, that is, stable state, in the environmental landscape. An example of such a shift is the introduction of a new fish species into a shallow lake ecosystem. The new species can change the environment within the lake by selectively feeding on planktivorous invertebrates. This increase in the death rate of planktivorous invertebrates can result in an increase in algae species within the lake that can lead to changes in water clarity, which influences plant growth and survival. The result is a very different community state in the lake than the one that occurred before the fish were introduced as a new component (ie, as a change in the environment) to the previous stable‐state community (Scheffer, Hosper, Meijer, Moss, & Jeppesen, 1993). Other examples include state changes in shallow‐water coastal ecosystems driven by sediment availability, sea‐level rise, and the relative strength of wind waves with respect to tidal currents (McGlathery et al., 2013) and state changes in a lake ecosystem associated with the intentional introduction of the brine shrimp, *Artemia sinica* (Jia, Anufriieva, Liu, Kong, & Shadrin, 2015).

## 2 STABLE VERSUS UNSTABLE SYSTEMS

As mentioned above, stable states are nontransitory over an ecologically relevant time scale. Here, we explore the idea that even systems that outwardly appear to be highly dynamic, that is, unstable, can be viewed within the framework of stable‐state theory if the appropriate “ecologically relevant” time scale is identified and used to define the system's community. Thus, within the normal bounds of environmental variation for a dynamic system, the stable‐state community would consist of a community amalgamation that includes all of the transitory communities that cycle in and out of existence as a dynamic system cycles through its natural range of environmental variability. The ecologically relevant time scale for these dynamic systems would therefore need to be defined by the length of time needed to complete an entire cycle through the system's normal range of environmental variation. For some systems, the ecologically relevant period can be relatively short (eg, tidal systems), for others it can be decadal (eg, prairie wetlands).

## 3 CONTINUOUS VARIATION AND THE CONCEPT OF MULTIPLICITY OF ECOSYSTEM ALTERNATIVE STABLE STATES

Ecosystem dynamics often occur as shifts along continuous gradients rather than as categorical jumps among alternative states (Capon et al., 2015). For freshwater wetlands, such shifts along continuous gradients form the framework of the Wetland Continuum described by Euliss et al., 2004. While ecosystems can realize smooth, continuous changes in response to changes in continuous environmental gradients, they can also reach tipping points in which they destabilize and transform into a new state. These two stages in the evolution and dynamics of ecosystems were identified as coherent and incoherent stages, respectively (Krasilov, 1986). Thus, ecosystems can exist for periods in a coherent stage in which they aggregate and transform resources, and cycle to an incoherent stage that creates opportunities for innovation and can lead to a shift to an alternative state in which the system may again exist in a coherent stage (Holling, 1973, 2001). A newer vision of ecosystem dynamics that incorporates both smooth changes in response to changes along continuous environmental gradients during coherent periods and large, abrupt, persistent shifts to alternative states in relation to tipping points during incoherent periods is incorporated into the concept of multiplicity of ecosystem alternative stable states (Biggs, Carpenter, & Brock, 2009; Holling, 2001; Walker, Holling, Carpenter, & Kinzong, 2004). This concept has been suggested a key scientific element upon which to base substantiable environmental management (Shadrin, 2018). We explore the concept of alternative stable states in unstable systems using the concept of multiplicity of ecosystem alternative stable states, and the highly dynamic wetlands of the North American Prairie Pothole Region (van der Valk, 2005) as our example system.

## 4 PRAIRIE‐POTHOLE WETLANDS

Prairie‐pothole wetlands are known for their highly variable temporal dynamics (Winter & Rosenberry, 1998). These inherently dynamic systems are not the type of systems that one would generally associate with concepts related to stable‐state theory. The plant, invertebrate, amphibian, bird, and fish communities in these prairie‐pothole wetlands continually transition through multiple states in response to sometimes decadal‐long climate oscillations that cyclically influence ponded‐water depth, permanence, and chemistry (Winter, 2003). Community changes in prairie‐pothole wetlands are driven by oscillations between periods of lower than average precipitation and periods of higher than average precipitation (van der Valk & Davis, 1978; Winter & Rosenberry, 1998). The changes in precipitation result in low (or absent) ponded‐water levels during periods of lower than normal water inputs and relatively high ponded‐water levels during periods of greater than normal inputs.

Changes in water depth and volume driven by variable precipitation patterns result in corresponding changes in the length of time that surface waters are ponded in a wetland, referred to as a wetland's “pond permanence” (Hayashi, Kamp, & Rosenberry, 2016). Water‐volume changes also have a marked influence on the chemistry of ponded water (especially salinity) through concentration and dilution effects during drought and deluge periods, respectively (LaBaugh et al., 2018). Unsurprisingly, the biotic communities of prairie‐pothole wetlands are also highly dynamic over time as they respond to changes in ponded‐water depths, permanence, and chemistry (Mushet et al., 2015). Community changes in prairie‐pothole wetlands in response to these wet/dry cycles are most readily observable in the plant communities (Stewart & Kantrud, 1972). However, changes also occur in the less visible invertebrate and vertebrate communities in response to not only changes in water depth, permanence, and salinity (Euliss et al. 2004), but also to changes in the primary producers, that is, the wetland plant and algal communities (Gleason, Bortolotti, & Rooney, 2018).

## 5 THE MARSH VEGETATION CYCLE

**Figure 2:**
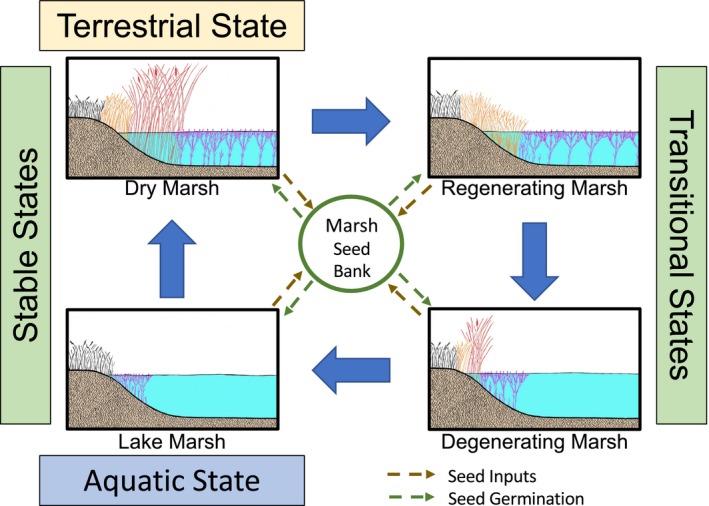
The prairie‐marsh cycle (van der Valk & Davis, 1978) depicting how prairie wetlands transition through various phases that include the dry‐marsh, regenerating‐marsh, degenerating‐marsh, and lake‐marsh. Marsh seed banks receive contributions from and provide recruitment to the plant communities within each of the various marsh phases. The dry‐marsh phase and lake‐marsh phase represent terrestrial and aquatic communities, respectively, and can be considered as stable states. The regenerating‐marsh and degenerating‐marsh phases are transitional states between the dry‐ and lake‐marsh states. (figure modified from van der Valk & Davis, 1978)

**Figure 3:**
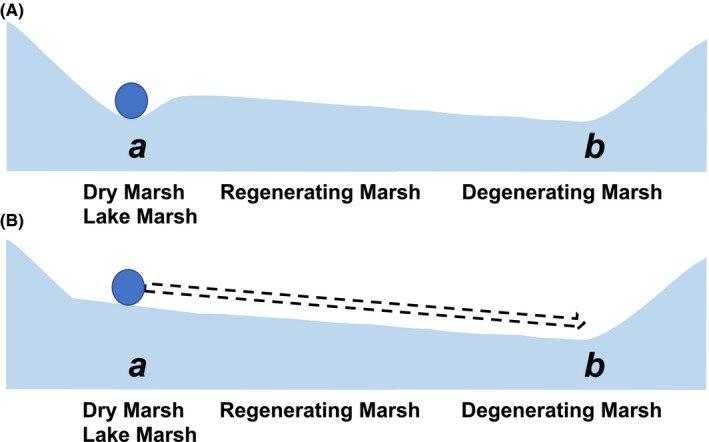
Ball‐and‐cup diagram depicting the prairie‐marsh cycle with domains of attraction (ie, stable states) located at *a* and *b*, that is, the dry‐marsh phase and lake‐marsh phase, respectively (A). If a wetland in the dry‐marsh phase (*a*) fills with water, the underlying environmental landscape changes (B) such that the plant and animal communities (represented by the ball) begin their journey through the regenerating and degenerating phases to the new domain of attraction (*b*) at the lake‐marsh phase. The communities will remain in this new state until the marsh once again dries and the cycle repeats

van der Valk and Davis (1978) illustrated the cyclical nature of prairie‐pothole wetland plant communities as they transition through phases identified as the dry‐marsh, regenerating‐marsh, degenerating‐marsh, and lake‐marsh phase (Figure 2). While it is common to consider all four phases to be transitional phases, it is also equally plausible to consider the dry‐marsh and lake‐marsh phases to be stable states with the regenerating‐marsh and degenerating‐marsh phases as transitional phases between these stable‐state endpoints (Figure 3). Thus, if the wetland were to remain dry, a stable‐state terrestrial community would persist. Likewise, if the wetland were to remain permanently ponded, a stable‐state aquatic community would persist. However, the regenerating‐marsh and degenerating‐marsh phases are clearly transitory, as their names suggest. It is the continuous environmental variability, primarily in the form of changing precipitation inputs, that results in the cycling of prairie‐pothole wetlands through the differing phases described by van der Valk and Davis (1978). However, while the dry‐marsh and lake‐marsh phases can be considered as alternative stable states, the continuous cycling of the meteorological conditions in the region results in wetlands rarely if ever spending a long enough time in either of these two phases for them to really be considered as nontransitory. Thus, while in theory these two end‐point phases could be considered to be the stable states of these dynamic systems, in reality the natural environmental variation of the region results in the dry‐marsh and lake‐marsh phases also being transitory in nature.

## 6 THE WETLAND CONTINUUM

**Figure 4:**
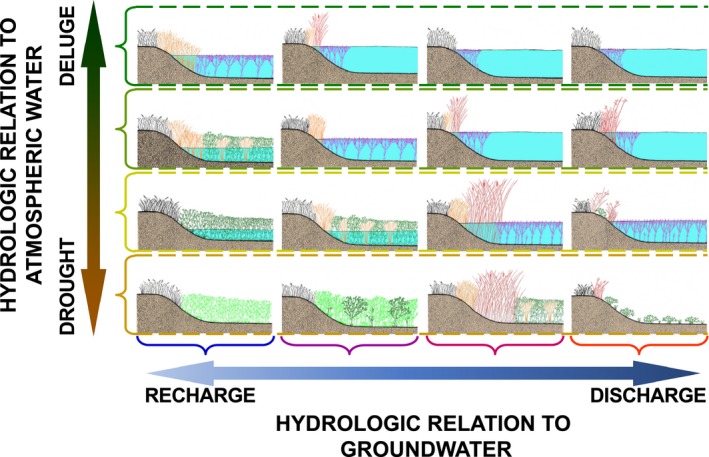
The wetland‐continuum framework (Euliss et al., 2004) showing how wetlands can occur along two environmental axes representing their “hydrologic relation to atmospheric water” and their “hydrologic relation to groundwater.” Wetlands toward the “recharge” end of the groundwater axis primarily lose water to the groundwater system and are very fresh. Wetlands toward the discharge end of the same axis receive discharge from groundwater and are highly saline. Plant and animal communities respond to the unique hydrological and geochemical conditions present in a wetland at any given point defined by these two axes. (figure modified from Euliss et al., 2004)

Another conceptual model describing the dynamics of prairie‐pothole wetlands is the wetland‐continuum framework (Figure 4) proposed by Euliss et al. (2004). Within the wetland continuum, the cycling of wetlands among the differing phases described by van der Valk and Davis (1978) is captured along a vertical, that is, atmospheric water, axis. The biotic communities of wetlands change along this axis as meteorological conditions change in terms of the amount of precipitation and snow meltwater entering a wetland. In contrast to the marsh cycle of van der Valk and Davis (1978), the wetland‐continuum framework includes a second dimension, along which the position of a wetland is determined by its hydrologic relation to groundwater. This second continuous gradient varies between recharge wetlands (wetlands that contribute water to but do not receive water from the groundwater system) and discharge wetlands (wetlands that receive water from but do not contribute water to the groundwater system). The majority of wetlands fall along the continuous gradient between these two extremes, where they both receive water from and discharge water to the groundwater‐flow system. Euliss et al. (2004) describe how the flushing of salts with groundwater recharge and the importation of salts with groundwater discharge result in wetlands toward the recharge end of the groundwater gradient being extremely fresh, while wetlands located toward the discharge extreme are highly saline.

The plant and animal communities present in wetlands at varying positions along the continuous recharge to discharge gradient respond differently to the continuous variation along meteorologically driven atmospheric‐water axis. Thus, while the marsh vegetation model (Figure 2) of van der Valk and Davis (1978) is principally focused on wetlands located near the central positions of horizontal (groundwater) axis of the wetland‐continuum model (Figure 4), wetland communities nearer the “recharge” and “discharge” extremes of the wetland‐continuum model also vary in response to changing atmospheric‐water inputs. These wetlands might not follow the typical dry‐marsh, regenerating‐marsh, degenerating‐marsh, and lake‐marsh progression of wetlands located more centrally along the wetland continuum's horizontal axis (Euliss et al., 2004); however, they still cycle between terrestrial states during drought and aquatic states during deluge.

## 7 THE STABLE STATE OF AN UNSTABLE SYSTEM

**Figure 5:**
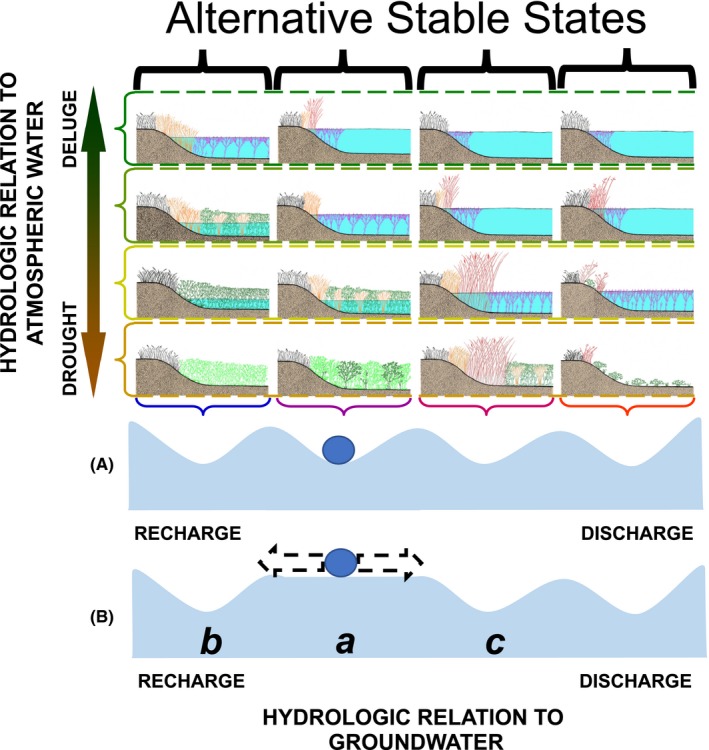
Ball‐and‐cup diagram depicting how the range of plant and animal communities at any position along the groundwater axis of the wetland continuum (Euliss et al., 2004) can form domains of attraction and therefore be considered as alternative stable states (A). However, changes in the underlying environmental landscape can cause shifts between alternative stable states. In the example shown in (B), the domain of attraction at position *a* in the wetland continuum has been altered due to changes in the underlying environmental landscape such that biotic communities are poised to shift to an alternative stable state (*b* or *c*)

While biotic communities of prairie‐pothole wetlands are continually changing in response to cyclical changes in meteorological conditions, the changes are predictable, and communities can be described at any point defined by the two axes of the wetland continuum and associated domains of attraction (Euliss et al., 2004). However, we postulate that rather than considering the dry‐marsh and lake‐marsh phases of prairie‐pothole wetlands to be the stable states (Figure 3), the entirety of the cycling of wetland communities in response to cyclical changes in meteorologically driven changes in ponded‐water depth, permanence, and salinity is in actuality the “stable state” of these highly dynamic systems (Figure 5). This view of a stable state that encompasses a system's range of cyclical variation is in line with the pulsing steady‐state paradigm put forth by Odum, Odum, and Odum (1995), but at an even longer time scale than the tidal or seasonal pulses they describe for coastal and inland wetland systems. It is also consistent with the ideas of nested, interacting, hierarchical scales described as a panarchy by Holling, Gunderson, and Peterson (2002).

While the surface expression of the plant and animal communities of prairie‐pothole wetlands may appear to be constantly changing, if the plant and invertebrate seedbanks in these wetlands (see van der Valk & Davis, 1978 and Gleason, Euliss, Hubbard, & Duffy, 2003, respectively) are considered as part of the biotic community, the overall stability of these dynamic systems is more apparent. Mushet, Solensky, and Erickson (2019) described this unexpressed diversity in wetlands as “dark diversity,” similar to the dark diversity described by Pärtel, Szava‐Kovats, & Zobel, 2011. However, rather than existing in other positions on the landscape as described by Pärtel et al., 2011, the dark diversity described by Mushet et al. (2019), exists in seedbanks and eggbanks within an ecosystem, awaiting more favorable environmental conditions before being more visibly expressed. Shadrin (2018) similarly described the resting stages of biota as a system's “sleeping” biodiversity. Thus, if the predictable variation of wetlands in terms of their biotic communities, as maintained through their dark or sleeping biodiversity, is considered to be the stable state for these systems, one can then begin exploring what their alternative stable states might be.

## 8 ALTERNATIVE STABLE STATES IN UNSTABLE SYSTEMS

The wetland‐continuum conceptual framework provides an ideal framework within which to explore the concept of alternative stable states in unstable systems. If the changes that wetland communities undergo in response to cyclical changes in atmospheric inputs (ie, the vertical axis of the wetland continuum) define the stable states of these systems (Figure 5A), transitioning to an alternative stable state would require the wetland to shift in its positioning along the wetland continuum's horizontal axis (Figure 5B). Thus, while a wetland ecosystem is responding along the continuous environmental gradient defined by the vertical axis, the system is in a coherent period that can last for decades. However, if a wetland enters an incoherent period, a horizontal shift can occur. At the new position along the horizontal axis, the biotic community enters another coherent period and once again varies predictably in response to the cyclical changes in atmospheric inputs. However, the community composition at any given position along the vertical axis would be different, as predicted by the wetland‐continuum concept of Euliss et al. (2004), than the community composition at that same position on the vertical axis if the wetland had remained at its previous position along the horizontal axis.

In the southern Prairie Pothole Region, we observed such a shift in wetland communities (McKenna, Mushet, Rosenberry, & LaBaugh, 2017). Several lines of evidence revealed that persistent increases in precipitation that started in 1993 resulted in changes to both surface and groundwater flows to prairie‐pothole wetlands. These lines of evidence included changes in pond numbers, ponded‐water depths, lake levels, stream flows, groundwater heights, soil‐moisture, waterfowl populations, and installation of subsurface tile drains in agricultural fields. In addition to altering wetland water levels and pond permanence, these changes were associated with a persistent build‐up of salts in the wetlands, which have been shown to lead to transitions to alternative states (Davis et al., 2003). This salinization of wetlands is consistent with a shift in the positioning of wetlands along the groundwater axis of the wetland continuum (Figure 5B; Mushet, McKenna, LaBaugh, Euliss, & Rosenberry, 2018). Similar changes in prairie‐pothole wetlands have been documented in the northern portion of the Prairie Pothole Region (Hayashi et al., 2016). Understanding that the stable state of dynamic systems can shift to an alternative stable state is key to developing conservation, mitigation, and adaptation strategies for these systems in which changes in how these systems function may be masked by their inherent instability, and therefore easily missed or misunderstood.

## 9 CONCLUSIONS

While stable‐state theory has typically not been applied to systems that undergo dynamic changes to variable environmental conditions, we show how the ideas presented within stable‐state theory can be applied to these dynamic systems if the normal range of community variation during a coherent period is considered to be the “stable state.” By doing so, one can then explore how community and environmental drivers, and tipping points during incoherent periods can work to cause state shifts that result in community changes outside of the bounds of the previous stable‐state community. Prairie‐pothole wetland ecosystems provide an example of shifting stable states within a highly dynamic system. The perspective gained by considering these dynamic systems in the context of stable‐state theory and the concept of multiplicity of ecosystem alternative stable states allow for an increased understanding of how they respond to changing community and environmental drivers during both coherent and incoherent periods. A better understanding of the domains of attraction and the community and environmental drivers that can push dynamic systems to alternative stable states will become increasingly important as changing climate and land‐use conditions across the globe threaten both stable and dynamic ecosystems without prejudice.

## CONFLICT OF INTEREST

None declared.

## AUTHOR CONTRIBUTIONS

DMM conceived and led the writing of the manuscript. DMM, OPM, and KIM all contributed substantially to the development of the ideas presented, contributed critically to drafts, and provided final approval for publication.

## Data Availability

This is a conceptual paper and did not involve the use of any new or previously unpublished data.
